# Multinodular Hydropic Leiomyoma in a 41-Year-Old Patient: A Case Report

**DOI:** 10.3390/jcm14217615

**Published:** 2025-10-27

**Authors:** Diana Xie Freire, Alissia Blumer, Teresa Teixeira da Silva, Sonali Düblin, Joachim Diebold, Ivo Fähnle-Schiegg

**Affiliations:** 1 Department of Gynecology and Obstetrics, Luzerner Kantonsspital, 6210 Sursee, Switzerland; teresa.teixeiradasilva@luks.ch (T.T.d.S.); sonymendis@gmail.com (S.D.); if@gynx.ch (I.F.-S.); 2 Departement Frauen und Kinder, Kantonsspital Baden, Im Ergel 1, 5404 Baden, Switzerland; 3 Department of Pathology, Luzerner Kantonsspital, 6000 Lucerne, Switzerland; alissia.blumer@luks.ch (A.B.); joachim.diebold@luks.ch (J.D.)

**Keywords:** hydropic leiomyoma, uterine fibroids, multinodular, perinodular hydropic degeneration, leiomyoma variant, case report

## Abstract

Uterine leiomyomas are a heterogenous group of benign mesenchymal tumours. While diagnosis is usually achieved through clinical assessment and pelvic ultrasound (PU), atypical subtypes are not as easily recognisable and can be mistaken for malignant tumours such as leiomyosarcoma or ovarian carcinoma. We describe the case of a 41-year-old patient who presented with increasing bulk symptoms, urinary frequency and growth of a hydropic leiomyoma (HLM) of the left lateral and posterior uterine wall that had been known for 10 years, confirmed with previous biopsy. The tumour filled the entire pelvic cavity in PU and was increasingly difficult to delineate; therefore an abdominal hysterectomy without oophorectomy was performed. Gross tissue examination showed an irregularly enlarged, asymmetric uterus with an intrauterine subserosal mass and an extrauterine papillary tumour arising from the right and posterior uterine wall. The tumour measured 20 × 17 × 10 cm in size. Numerous smooth muscle nodules were observed within the uterus and extending into the extrauterine component in a continuous transition, exhibiting a benign, bland appearance. The nodules were separated by abundant edematous connective tissue with increased vascularization. Histopathological analysis revealed low mitotic activity with no evidence of nuclear atypia, pleomorphism, or necrosis. Immunohistochemical staining confirmed the diagnosis of a benign smooth muscle tumour. Our findings confirm a rare, benign smooth muscle neoplasm with both intrauterine and extrauterine involvement, and add to the existing literature regarding presentation, diagnostic and therapeutic challenges associated with HLM.

## 1. Introduction

Uterine leiomyomas, also known as uterine fibroids, are amongst the most common benign gynecologic tumours, affecting up to 51% of premenopausal women. Their growth is associated with hormonal factors and their incidence is more common with increasing age. Other risk factors such as family history, environmental factors and race are also thought to play a role [1,2,3].

It is estimated that only a quarter of leiomyomas lead to symptoms, such as heavy menstrual bleeding, pelvic pain, infertility, urinary symptoms or dyspareunia [4]. The intensity and type of symptoms depend on the size, amount and location of myomas.

Diagnosis is usually made through physical examination and pelvic ultrasound (PU) alone. Sometimes this basic imaging must be complemented by further examinations, such as sonohysterography, hysteroscopy [5,6,7,8] or, in complex cases, by magnetic resonance imaging (MRI). If malignancy is suspected, computer tomography (CT) scans and positron emission tomography (PET) are usually performed to help differentiate between leiomyomas and leiomyosarcomas or ovarian tumours, even though this has not been proven to be sufficiently reliable [9,10,11,12,13]. It is not always possible to obtain histopathological confirmation during the diagnostic stage, which increases the difficulty of decisions regarding therapeutic management.

Therapeutic options range from symptom-oriented and hormonal approaches, uterus- preserving interventions such as myomectomy by laparoscopic or hysteroscopic resection, uterine artery embolization as well as radiofrequency ablation, to radical approaches in the form of hysterectomy [14]. The patient’s desire for future pregnancies must be considered when discussing these options.

Due to the advances in the fields of genomics and molecular pathology, an increasing number of histological subtypes and variants have been found in the heterogeneous group of uterine leiomyomas, which is also reflected in the 2014 WHO classification of mesenchymal uterine tumours [15]. Atypical variants remain rare, comprising about 3% of all cases [16]. In this case report, we present a subtype of uterine leiomyomas, namely multinodular hydropic leiomyomas (HLM), whose incidence remains unknown due to its rarity [17,18], and discuss associated challenges in imaging, histopathological assessment, management and postoperative outcomes.

## 2. Case

We present the case of a 41-year-old Caucasian secundipara who had been diagnosed with a leiomyoma 10 years prior. At initial diagnosis through PU, the mass was located in the left lateral uterine wall, measured around 4 to 5 cm and resembled a typical leiomyoma. Personal and family history was negative for gynaecological carcinomas. The remaining past medical and surgical history was unremarkable. Expectant management was pursued initially.

**Figure 1:**
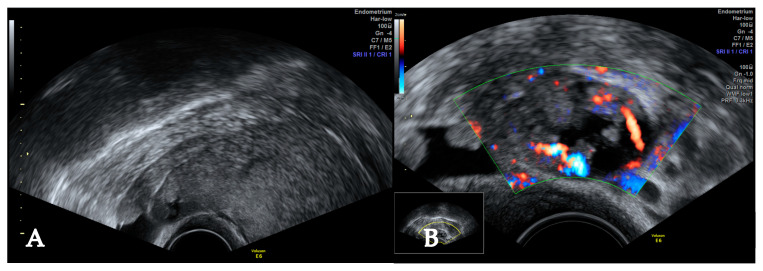
Pelvic ultrasound (PU) at the first consultation. Uterine mass suggestive for leiomyoma, reaching from the left lateral wall to the fundus (**A**). Doppler ultrasound shows a highly vascularized cystic, papillary structure attached to the mass (**B**).

The patient was 37 years old when she was first referred to our hospital group for a second opinion regarding increasing symptoms. She complained of urinary frequency and heavier menstrual bleeding since her first vaginal birth a year prior. PU showed an enlarged uterus with deviation to the right and a homogenous mass of around 10 × 6 cm on the left lateral wall of the uterus from the insertion point of the left uterine artery to the fundus with protrusions into the pouch of Douglas. The ventral, fundal, subserous junction points of the uterus to the mass showed atypical, cystic, papillary structures with increased vascularization (Figure 1). A PET as well as a CT scan of the abdomen and thorax were performed in order to distinguish between a possible leiomyosarcoma and they showed no suspicion of malignancy. Since the patient was still open to trying for a second pregnancy, watchful waiting was agreed upon.

**Figure 2:**
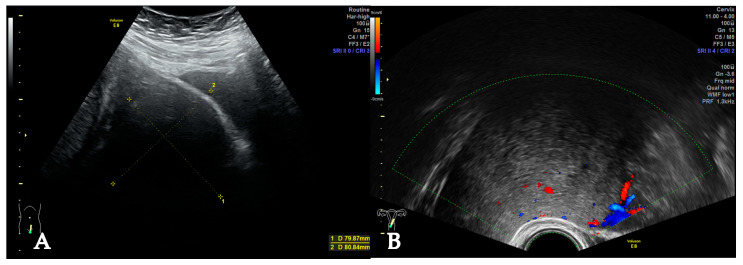
Uterine mass during pregnancy. Abdominal ultrasound at 38 weeks (**A**). Ultrasonographic findings were suggestive of deviation of the labour canal, especially as seen in PU (**B**). b—bladder, m—mass.

At the next consultation a few months later, she had conceived spontaneously. The first and second trimester were free of complications; the leiomyoma remained stable in size. However, at 38 weeks 4 days of gestation, the patient presented at our department with severe itching; cholestasis of pregnancy was diagnosed. The myoma was reevaluated as well due to worsening pelvic pain. Abdominal ultrasound (AU) and PU were suggestive of a slight enlargement of the myoma to 11 × 10 × 10 cm as well as a deviation of the cervix to the right. (Figure 2). We recommended early delivery due to cholestasis of pregnancy via cesarean section (CS) due to obstruction of the labour canal.

**Figure 3:**
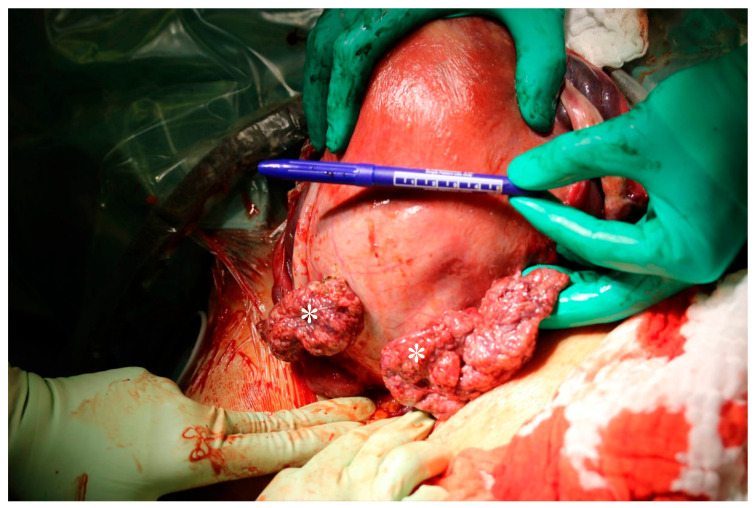
Intraoperative findings during cesarean section. Papillary structures (*) on the posterior uterine wall.

CS was performed at 38 weeks 5 days of gestation without any complications. The subserous myoma, measuring around 10 cm, contained attached macroscopic papillary structures in the posterior uterine wall, which were biopsied intraoperatively (Figure 3). Histology confirmed a multinodular HLM.

**Figure 4:**
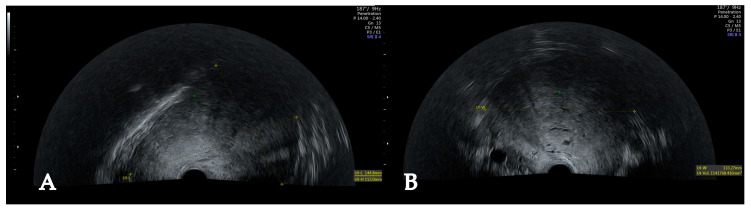
Growth progression of the leiomyoma. Increasing difficulty to delineate the tumour in sagittal (**A**) and transversal view of PU (**B**).

Over the following two and a half years, the patient was examined in regular intervals. She remained asymptomatic and the myoma showed no signs of growth progression. When she turned 41 years old, she developed bulk symptoms, especially involving the bladder, as well as worsened urinary frequency and dysmenorrhea. The myoma had grown to 13 × 13 × 15 cm and PU and AU were increasingly difficult to interpret, as the mass filled the entire pelvis (Figure 4). She was counselled for abdominal hysterectomy, since fertility-sparing options were no longer desired and the rapidly growing mass required definitive treatment. Informed consent was obtained.

**Figure 5:**
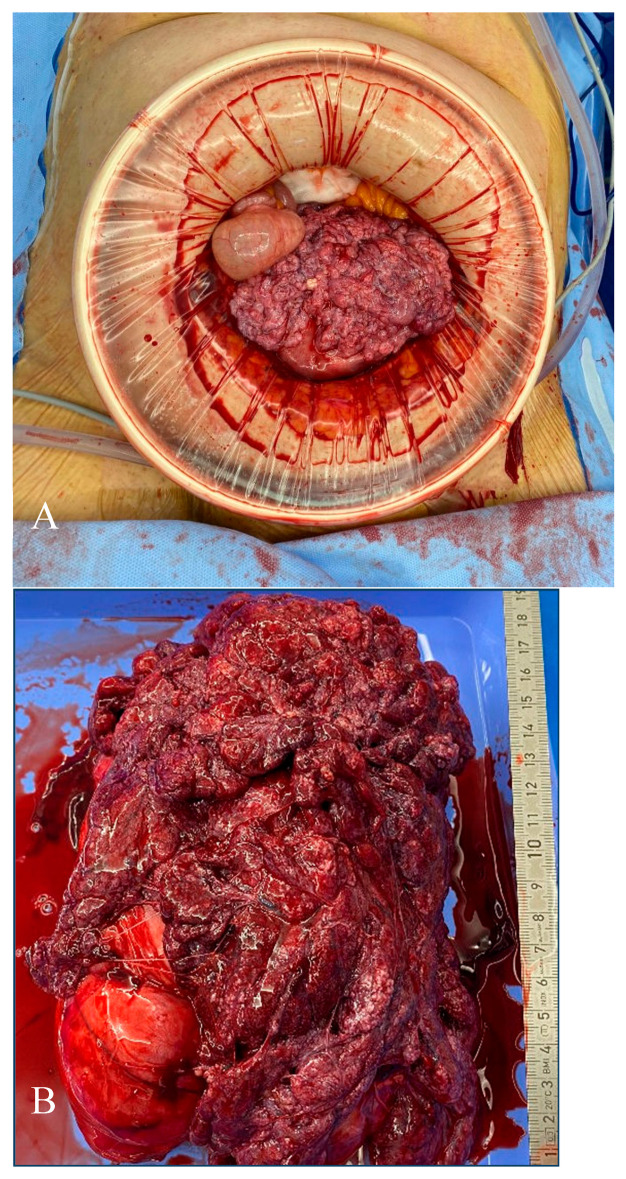
Intraoperative findings and macroscopy of the uterus. Macroscopic appearance of the uterus in situ (**A**) and after hysterectomy before fixation from a frontal (**B**) perspective.

Macroscopic examination of the uterus was unusual upon entering the peritoneal cavity, with cauliflower-like tissue protruding from the posterior uterine wall. Despite the past benign histology, a leiomyosarcoma could not be clinically ruled out (Figure 5), although on inspection, the peritoneal lining as well as all other intra-abdominal organs appeared normal and free of carcinomatosis. Several myomas in the left parametrium enlarged the uterus. The hysterectomy was complicated due to the inability to fully mobilise the uterus on the left side and therefore increased difficulty in incising the cervicovaginal junction due to the myomas. Ultimately, this was achieved by opening the vagina from the right side and incising towards the left until the uterine artery was clamped and cut through the vaginal opening. Cumulative intraoperative blood loss totaled 900 millilitres.

The patient recovered well despite increased pain during the first night, which required the administration of intravenous opiates. The haemoglobin value was at 110 g/L on the second postoperative day and AU was performed to rule out hydronephrosis before discharge.

**Figure 6:**
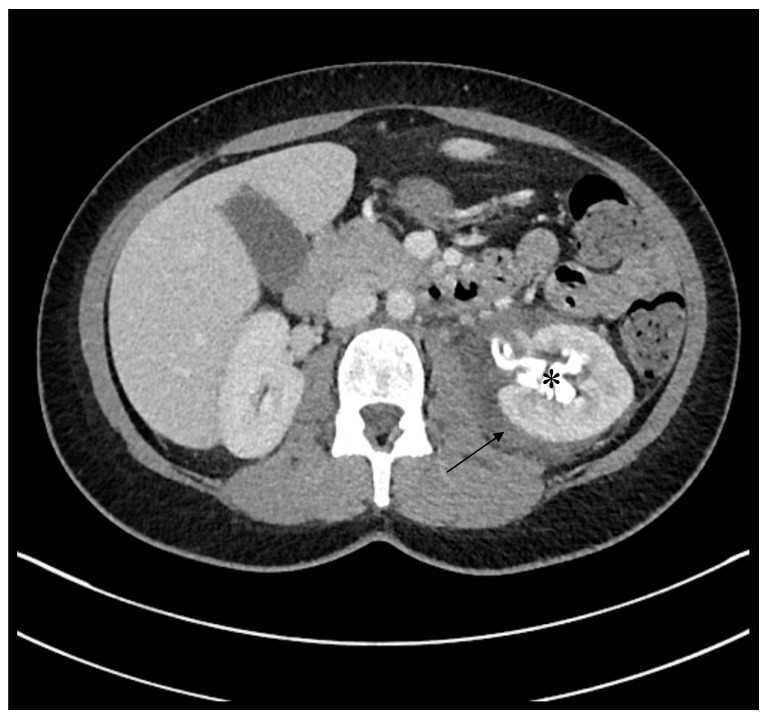
Postoperative computer tomography (CT) scan. Perirenal free fluid (arrow) as well as a leakage of contrast agent is seen exiting the left renal pelvis (*).

Two days after being discharged, she was admitted to the emergency room with strong flank pain on the left side. Clinical examination was not suggestive of pyelonephritis, but the estimated glomerular filtration rate (eGFR) was reduced with a rate of 53 mL/min. The CT scan showed a rupture of the left renal pelvis as well as an abrupt distal ending of the left ureter with no clear cause of obstruction (Figure 6). The patient underwent emergency pigtail catheterization of the left ureter and retrograde pyelography which showed an injury to the distal ureter. Ureteroneocystostomy in Politano-Leadbetter and Psoas-Hitch technique was performed, from which the patient recovered quickly. eGFR returned to >90 mL/min before the patient was discharged postoperatively after five days.

The patient presented to the gynecologic follow-up consultation almost two months later with complaints of urinary urgency, but no other symptoms. The pigtail catheter had already been removed. Clinical examination showed fully healed colpotomy and abdominal scars. The AU und PU of the kidneys, bladder and pelvis were normal. The patient was referred to the urologists for ongoing care regarding urinary urgency as well as bladder pain, which we suspected was due to the psoas hitch.

## 3. Histopathology

**Figure 7:**
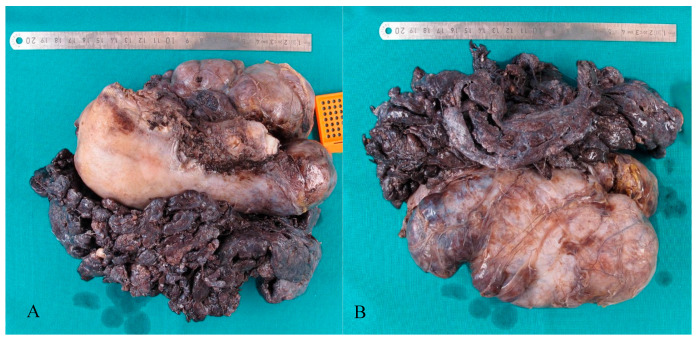
Hysterectomy specimen after fixation. Macroscopic appearance of multinodular HLM, displaying the exophytic, papillary fraction on the right and posterior uterine wall, as well as the nodular fraction on the posterior and left uterine wall. (**A**) Ventral view. (**B**) Dorsal view.

The pathological tissue examination of the hysterectomy specimen revealed a multinodular deformation in the cervix and corpus area, with a maximum diameter of 10 cm. Additionally, an exophytically growing, papillary tumour with a brown to livid colour was observed at the lateral and posterior corpus site, which contained several solid, white nodular components (Figure 7). The cut surfaces of both the intrauterine and extrauterine tumour areas exhibited a similar appearance, characterized by a white to brown coloration with a cribriform pattern, organized into numerous nodules of varying sizes and showing discharge of serous fluid. The tumour in the myometrial tissue of the uterus was poorly demarcated. Furthermore, small focal adenomyosis was observed, without any association with the smooth muscle neoplasia. Additionally, two normally structured fallopian tubes were identified, each with attached paratubal cysts, the largest of which measured up to 2.5 cm in diameter.

**Figure 8:**
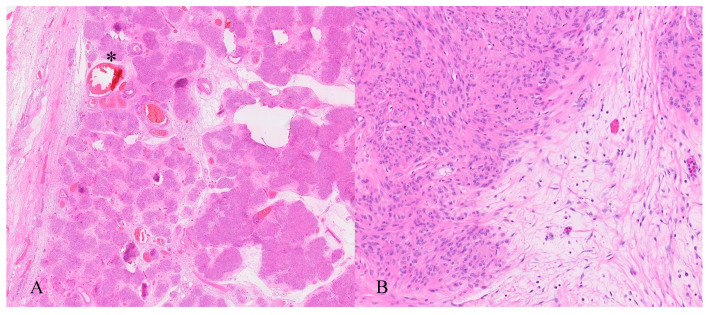
Histopathology. (**A**) Subserosal leiomyoma localized in the cervix with a multinodular appearance, arranged as fascicles of smooth muscle and separated by abundant watery-edematous stroma and containing congested vessels (*). (**B**) High magnification microscopic image: The smooth muscle cells are arranged in a swirling pattern. The cells are bland, spindle-shaped to oval, with no nuclear atypia or increased mitotic activity. The connective tissue between the muscle bundles appears hydropic, with few fibroblasts.

**Figure 9:**
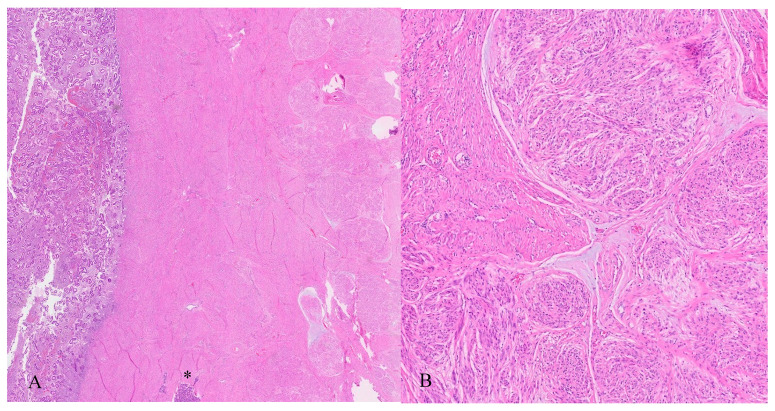
Histopathology. (**A**) HLM dissecting the myometrium of the uterus (right side of image). Intact secretory endometrium (left side). Adenomyosis uteri (*). (**B**) High magnification showing the HLM dissecting normal myometrial smooth muscle fascicles as well as hydropic changes within the myometrium.

**Figure 10:**
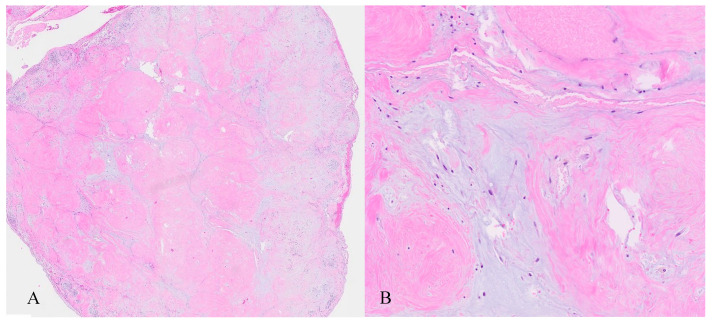
Histopathology. (**A**) Solid portion of the exophytic component of the multinodular HLM with pronounced hyalinization. (**B**) Higher magnification showing hyalinized smooth muscle fibre bundles separated by hypocellular, watery stroma.

**Figure 11:**
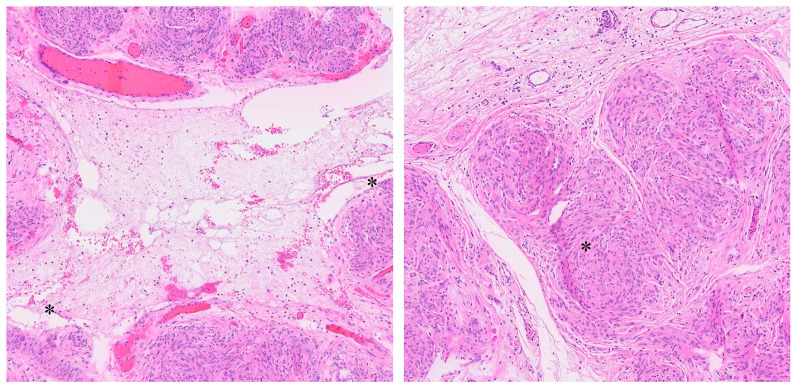
Histopathology. Smooth muscle nodules with perinodular hydropic degeneration with artefactual retraction of the intervening stroma (*), creating gaps that may mimic vascular infiltration.

**Figure 12:**
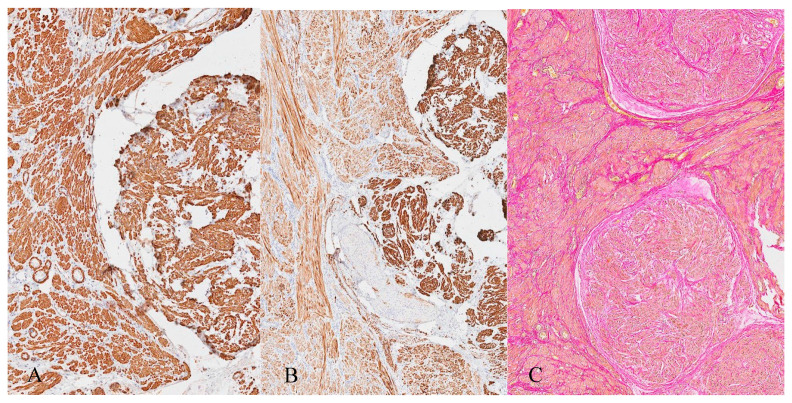
Immunohistochemical and special stains. (**A**) Actin is strongly positive in the myometrium and leiomyoma nodules. (**B**) Desmin is positive in myometrium and strongly positive in leiomyoma nodules. (**C**) EvG stain for the visualization of muscle tissue and blood vessels.

The neoplastic lesion predominantly consisted of spindle-shaped cells with round to oval nuclei arranged in nodular fascicles, exhibiting a swirled, disorganized pattern (Figure 8). These nodules were separated by abundant edematous, hypocellular stroma (perinodular hydropic degeneration) [17] and displayed an infiltrative growth pattern, with the tumour extending into the surrounding myometrium and causing tissue dissection (Figure 9). Some nodules in the exophytic tumour compound, corresponding to the macroscopically more solid, whitish parts, showed increased hyalinization (Figure 10). A subset of the nodules exhibited perinodular retraction of the hydropic fibroconnective tissue, which may have resembled intravascular infiltration (Figure 11). Immunohistochemical analysis revealed that the neoplastic cells were positive for smooth muscle antigen, desmin, and caldesmon (Figure 12). CD34 showed no endothelial cells delineating individual tumour nodules. No lymphangiosis was observed with D2-40. Tumour cells were negative for CD10.

## 4. Discussion

The pathogenesis of HLM is still poorly understood. Advanced analyses like molecular and genetic testing have been described in a few studies, but occurrences remain rare, which hinder new insights. Table 1 provides an excerpt of the overview on HLM case reports.

### 4.1. Diagnostic Challenges

Imaging of HLM poses a challenge, as it is often inconclusive. Some features of HLM, such as rapid growth and complex cystic structures, may be mistaken for other benign or malignant tumours of the ovary or leiomyosarcoma [17,19,20,21,25]. Pelvic ultrasound may not reveal characteristic features such as watery edema [22,23], while MRI provides better tissue contrast but cannot definitively exclude malignancy before surgical intervention [23,24,26]. Although diffusion-weighted imaging and the “split fibre” sign have shown promise [27], securing histopathology in a timely manner remains the safest and most reliable approach [19,22].

### 4.2. Histopathology and Differential Diagnosis

In complex and rare cases such as ours, histopathological examination can be challenging. Table 2 describes the antibodies used in the analysis of our specimen to secure the diagnosis and rule out other possible tumour entities.

This hydropic multinodular leiomyoma exhibits distinct perinodular hydropic degeneration, which divides the tumour into numerous smooth muscle nodules separated by watery, edematous connective tissue, creating its characteristic multinodular pattern. This degeneration can resemble intravascular infiltration or intravascular leiomyomatosis, especially when there is severe degeneration with retraction [17,28,29,30,31,32]. Intravenous leiomyomatosis refers to the growth of smooth muscle cells within blood vessels, extending beyond the uterus or beyond a coexisting uterine leiomyoma [33]. Histologically and immunohistochemically, these tumours correspond to benign leiomyomas [34,35] and are lined by endothelium [36,37]. They dilate and compress vessel walls and may frequently exhibit hydropic changes, hyaline degeneration, and angiomatous alterations [33,34,37]. Leiomyomas with vascular invasion are distinguished by their microscopic vascular infiltration confined within the boundaries of a leiomyoma [38,39]. Intravenous leiomyomatosis can then be ruled out by immunohistochemical staining of endothelial cells (i.e., CD34).

Leiomyoma with bizarre nuclei and fumarate hydratase-deficient leiomyoma are excluded due to the absence of atypical/bizarre tumour cells [32]. Cellular leiomyoma, characterized by increased cellularity compared to the surrounding myometrium, is ruled out as the leiomyoma described in our case shows normal cellularity. The mitotically active leiomyoma subtype demonstrates significantly more mitoses, with 2.5–6 mitoses/mm^2^ than the leiomyoma described here [32].

Unlike the leiomyoma described in this case report, myxoid leiomyoma is well circumscribed, hypocellular, and contains abundant myxoid stroma. In contrast, hydropic leiomyoma presents with protein-rich, pale to eosinophilic stroma, which can be distinguished from myxoid stroma based on histomorphology [32]. Another differential diagnosis is myxoid leiomyosarcoma, which is characterized by cytologic atypia, tumour cell necrosis, and increased mitotic activity [40]. The absence of atypia, low mitotic activity (1%), lack of nuclear atypia, pleomorphism, necrosis and myxoid stroma ruled it out.The tumour may show a vaguely fascicular or nodular growth pattern; in our case, multiple well-defined smooth muscle nodules were visible.

Smooth muscle tumours of uncertain malignant potential (STUMP) are also excluded as a differential diagnosis, since at least one criterion for the diagnosis of leiomyosarcoma must be met: either nuclear atypia with 2–4 mitoses/mm^2^, tumour cell necrosis alone, or more than 6 mitoses/mm^2^ [41].

Another differential diagnosis for HLM is low-grade endometrial stromal sarcoma [42]. Low grade endometrial stromal sarcoma also shows an intramural dissecting growth pattern with an abrupt transition between the tumour and the myometrium. The tumour cells are densely arranged in cellular islands but are organized in a sheet-like pattern instead of a fascicular pattern. The nuclei are ovoid to spindle-shaped, and hydropic degeneration is uncommon. A diffuse vascular network of arterioles and capillaries is present and vascular invasion can be prominent. Cytologic atypia is minimal, mitotic activity is generally low, and necrosis is not consistently observed. Common variant features include smooth muscle differentiation [42,43]. The architectural features, the absence of CD10 expression and lymphovascular invasion excluded this diagnosis.

Diffuse leiomyomatosis, characterized by poorly circumscribed, merging hypercellular smooth muscle nodules that symmetrically replace the myometrium [44], is unlikely, as the described leiomyoma shows well-defined, asymmetrically distributed nodules with extrauterine extension.

Dissecting leiomyoma also presents with smooth muscle nodules that infiltrate the myometrium irregularly, and hydropic changes may also be observed. If the tumour extends beyond the uterus, it is referred to as a cotyledonoid leiomyoma [30]; however, both HLM and dissecting leiomyomas can exhibit exophytic components. One approach to distinguish these entities is to determine the predominant tumoral component [17].

### 4.3. Management

Expectant management was initially pursued due to mild symptoms, benign biopsy and the patient’s desire for fertility-preserving treatment. However, gradual growth, increasingly inconclusive imaging, and extrauterine expansion led to surgical intervention. Preoperative histopathology is rarely available in similar cases, as imaging alone often leads to a suspicion of malignancy and radical surgical approaches [18,19,21,23]. In our case, due to prior benign histology, a simple abdominal hysterectomy without oophorectomy was discussed with the patient. Although our initial intraoperative macroscopic findings were not consistent with typical benign leiomyomas, malignancy did not appear likely due to the lack of ascites and carcinomatosis, therefore the extent of surgery was not altered.

### 4.4. Complications

Although the renal pelvis rupture was initially reported as having no clear cause, comparison with existing literature suggests plausible mechanisms for ureteral injury (UI) [45]. In our case, UI most likely occurred during clamping of the left uterine artery, either through thermal injury or complete transection, as surgical dissection on that side was difficulted by the distorted pelvic anatomy due to tumour bulk. AU on the second postoperative day did not detect hydronephrosis, which emphasizes the need for heightened intra- and postoperative vigilance, as UI is often diagnosed with a delay of 7 to 10 days postoperative. The intraoperative blood loss was also elevated at 900 mL, but comparable with existing literature suggesting a higher risk of blood loss > 400 mL for benign abdominal hysterectomy, especially in the case of uterine fibroids [46]. The patient did not develop symptomatic anemia.

### 4.5. Limitations

As a single case report, our study has limited generalizability; the rare occurrence and unknown true incidence of this disease contribute to this fact. The availability of a prior benign biopsy justified expectant management, but this is not usually feasible in similar cases. At the time this case report was written, no data on long-term follow-up was available yet, especially regarding recovery from the ureteral injury. We also did not perform molecular analysis, which might have provided additional insights. Nevertheless, individual contributions to the literature regarding atypical leiomyoma presentation and histology are helpful to fill gaps and encourage further studies as well as raise awareness for these rare, but possible differential diagnoses, so that the best diagnostic and therapeutic decisions can be made available to patients.

Our experience highlights the need for individualized management of atypical leiomyomas as well as the importance of expert surgical planning in complex cases, particularly when diagnostic or surveillance imaging is inconclusive. Our case contributes with additional management during pregnancy and the complication of iatrogenic ureteral injury.

## 5. Conclusions

Uterine mesenchymal tumours represent a heterogeneous group. While atypical leiomyoma variants are estimated to account for up to 3% of all cases, the exact incidence of multinodular HLM remains unknown. The clinical and radiological presentation of HLM can closely mimic malignant tumours, which can only be ruled out through surgery and histopathological evaluation. Our case adds to the limited literature on this particular subtype, especially regarding its extrauterine expansion, growth progression during pregnancy and surgical complications. Careful pre- and intraoperative management is essential for optimal treatment of such rare variants.

## Figures and Tables

**Table 1:** Excerpt of reported cases of hydropic leiomyoma (HLM) in the literature.

Author, Year	Age (Years)	Tumour Size (cm)	Presentation	Diagnostics	Management	Outcome
El Ghali et al., 2022 [18] 2 cases	46	16 and 26	Pelvic pain, menorrhagia and progressive abdominal distension and menorrhagia	MRI: solid and cystic, intermediate T2 intensity and high T2 intensity, areas of necrosis	Hysterectomy	Uneventful
Akkour et al., 2021 [19]	32	33	Abdominal distension, pelvic pain, suspicious for ovarian malignancy or leiomyosarcoma	MRI: cystic, huge abdomino-pelvic masses	Myomectomy	Intraoperative hemorrhage of 6L, blood transfusion, recovery uneventful
Erfani et al., 2024 [20]	31	35	Abdominal distension, suspicious for ovarian malignancy, elevated CA-125 levels	CT/MRI: inconclusive large pelvic tumour	Myomectomy	Not disclosed
Zou et al., 2024 [21]	45	20	Elevated CA-125, Pseudo-Meigs syndrome, suspicious for ovarian malignancy or leiomyosarcoma	CT: suspicion of ovarian malignancy, hydrothorax, ascites	Hysterectomy	Uneventful
Horta et al., 2015 [22]	35	20	Diffuse abdominal bloating	MRI: T2 hyperintense, cystic	Hysterectomy	Uneventful
Yamaguchi et al., 2024 [23]	49	20	Abdominal distension, suspicious for ovarian malignancy	MRI: T2 hyperintense, cystic, ovaries not sufficiently assessable	Hysterectomy	Uneventful
Lameira et al., 2022 [24]	54	18	Rapidly growing abdominal mass, suspicious for malignancy	MRI: T2 hyperintense, hydropic	Hysterectomy	Uneventful

**Table 2:** Antibody panel used for tumour analysis.

Markers	Staining Pattern	Expression	Interpretation
CD34	Membranous stain	Positive in vascular endothelial cells	Endothelial differentiation
D2-40	Membranous stain	Positive in lymphatic endothelial cells	Lymphatic endothelial marker for the exclusion of lymphangiosis
Aktin	Cytoplasmic stain	Strongly positive	Expressed in tumours with myogenic differentiation; in endometrial stromal sarcoma only positive in areas of smooth muscle differentiation
Caldesmon	Cytoplasmic stain	Strongly positive	Expressed in tumours with myogenic differentiation; in endometrial stromal sarcoma only positive in areas of smooth muscle differentiation
Desmin	Cytoplasmic stain	Strongly positive	Expressed in tumours with myogenic differentiation; in endometrial stromal sarcoma only positive in areas of smooth muscle differentiation
CD10	Diffuse cytoplasmic or membranous	Negative	Positive in endometrial stromal sarcoma (sensitivity 91%, specificity 45%)
Ki-67	Nuclear stain	1%	Very low proliferation index which indicates low tumour malignancy

## Data Availability

The original contributions presented in this study are included in the article. Further inquiries can be directed to the corresponding author(s).
